# Multiple liver metastases originating from synchronous double cancer of neuroendocrine tumor and rectal cancer: a case report

**DOI:** 10.1186/s40792-020-0800-9

**Published:** 2020-02-13

**Authors:** Sachie Omori, Noboru Harada, Takeo Toshima, Kazuki Takeishi, Shinji Itoh, Toru Ikegami, Tomoharu Yoshizumi, Masaki Mori

**Affiliations:** The Department of Surgery and Science, Graduate School of Medical Sciences, Kyushu University, Maidashi 3-1-1, Higashi-ku, Fukuoka, 812-8582 Japan

**Keywords:** Neuroendocrine tumor (NET), Carcinoid, Mesentery, Liver metastasis, Synchronous double cancer

## Abstract

**Background:**

Neuroendocrine tumor (NET) is a relatively rare tumor and can develop in almost any organ, but primary mesenteric NETs are extremely rare. In addition, liver metastases from synchronous double cancer of neuroendocrine tumor graded as G1 and second primary malignancies (SPMs) have never been reported before. We herein report a case of multiple liver metastases from synchronous double cancer of NET (G1) at the ileal mesentery and rectal cancer.

**Case presentation:**

A 66-year-old man was identified as having tumors in the rectum and the ileal mesentery by computed tomography (CT). He underwent laparoscopic low anterior resection for rectal cancer and biopsy of the ileal mesentery lymph node and was diagnosed with rectal cancer as pT3 pN1 cM0 (stage IIIB) and NET (G1) of the ileal mesentery. He received oxaliplatin and capecitabine (XELOX) for 3 months as adjuvant chemotherapy for rectal cancer. The NET (G1) of the ileal mesentery was low grade and had not expanded at follow-up. A CT scan performed 4 years after the surgery indicated multiple liver metastases. All the metastases had the same findings on CT and magnetic resonance imaging (MRI). Thus, the patient underwent the first stage of modified associating liver partition and portal vein ligation for staged hepatectomy (modified ALPPS), comprising partial hepatectomies of segments 3 and 4, ligation of the right branch of portal vein, and hepatic partition on the demarcation line, followed by the second stage of modified ALPPS (right lobectomy). Histopathological findings revealed that the 14 nodules were metastatic liver tumors of rectal cancer and the 2 nodules were liver metastases of the NET (G1).

**Conclusions:**

Our findings suggest that synchronous double cancer of NET and gastrointestinal cancer may be indistinguishable in preoperative images. However, curative resection, precise pathological diagnosis, and adequately adjusted treatment may result in a better prognosis.

## Background

Neuroendocrine tumor (NET) is a relatively rare tumor and may occur in almost any organ [1]. Most NETs occur in the gastrointestinal tract, pancreas, and bronchopulmonary system [2], and primary mesenteric NET is extremely rare. Additionally, the development of second primary malignancies (SPMs) in patients with gastrointestinal NETs (GI-NETs) is a well-described phenomenon in Western countries [3], but there are few reports of SPM with GI-NET in Japan, and liver metastases from synchronous double cancer of NET graded as G1 and SPM have never been reported.

We herein report a case of multiple liver metastases from synchronous double cancer of NET (G1) in the ileal mesentery and rectal cancer.

## Case presentation

**Fig. 1:**
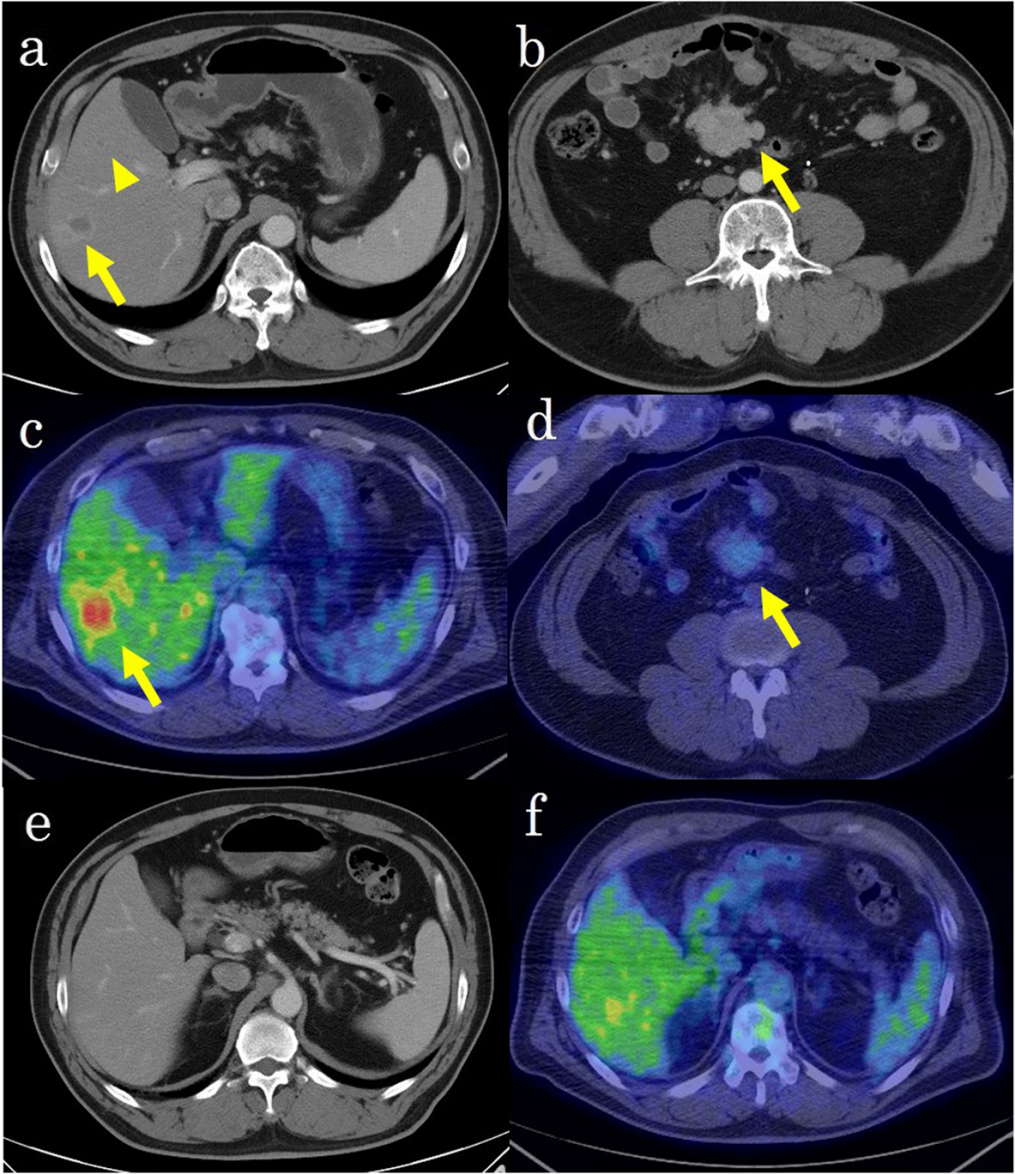
Contrast-enhanced computed tomography (CT) and [18F]-fluorodeoxyglucose positron emission tomography (FDG-PET) images 1 year after abdominoperineal resection. **a** Contrast-enhanced CT showed two tumors in segment 5 (arrow and arrowhead) that were enhanced in the arterial phase. **b** Contrast-enhanced CT showed a 47-mm tumor in the ileal mesentery (arrow) that was enhanced in the arterial phase. **c** On FDG-PET, accumulation of [18F]-FDG was found in segment 5 (arrow) tumor. **d** On FDG-PET, only a little accumulation of [18F]-FDG was found in the ileal mesenteric tumor (arrow). **e** Contrast-enhanced CT showed no tumor in segment 6 that was enhanced in the arterial phase. **f** On FDG-PET, no accumulation of [18F]-FDG was found in segment 6

**Fig. 2:**
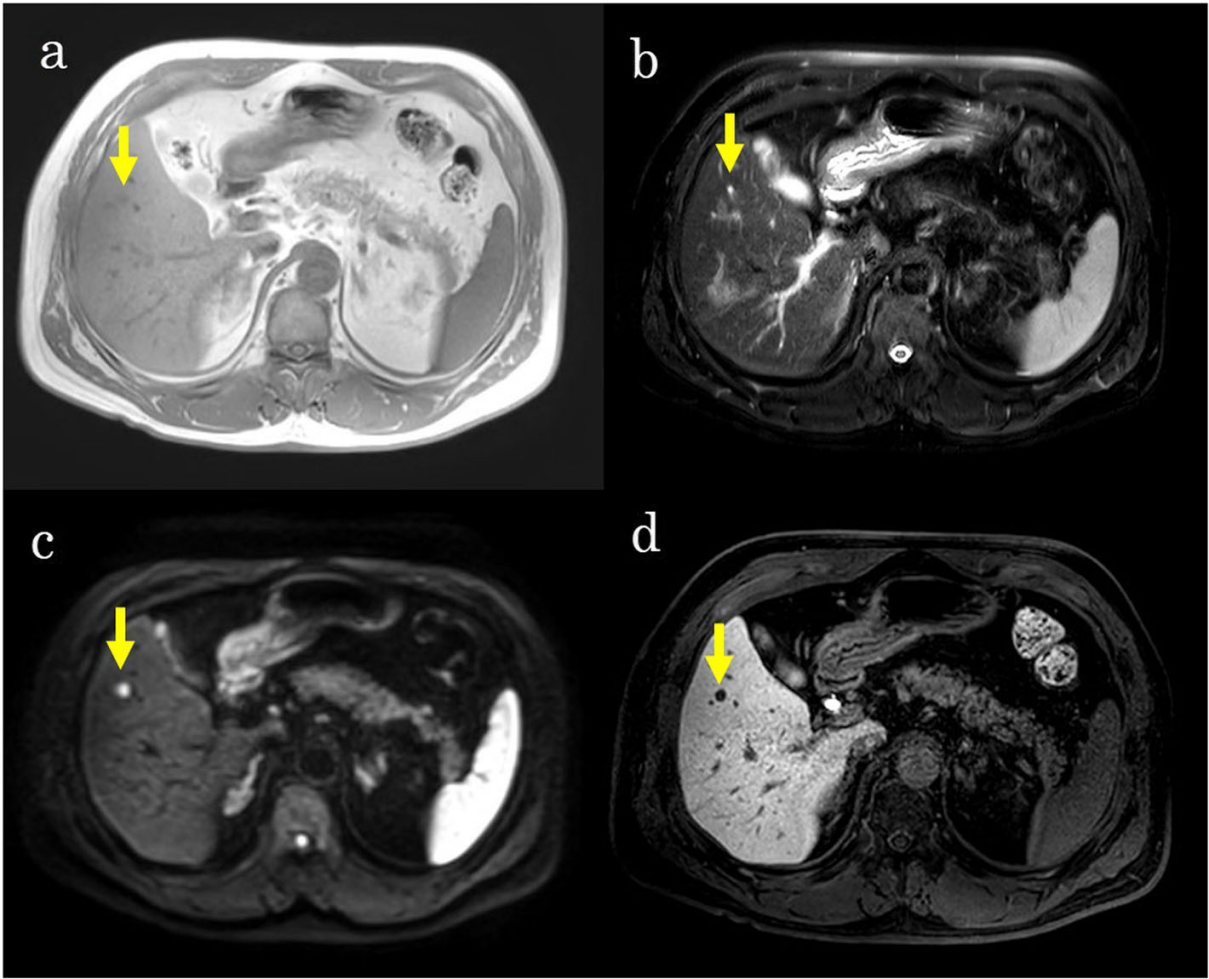
Magnetic resonance imaging (MRI) 1 year after abdominoperineal resection. **a** The tumor in segment 6 of the liver (arrow) showed low intensity on T1-weighted images. **b** The tumor in segment 6 of the liver (arrow) showed high intensity on T2-weighted images. **c** The tumor in segment 6 of the liver (arrow) showed high intensity on diffusion-weighted images. **d** The tumor in segment 6 of the liver (arrow) showed low signal intensity on gadolinium-ethoxybenzyl-diethylenetriamine pentaacetic acid-enhanced MRI (EOB-MRI)

A 66-year-old Japanese man was referred to our hospital because of a rectal tumor. He had a history of radical prostatectomy for prostate cancer and transurethral bladder tumor resection. Abdominal computed tomography (CT) showed a tumor of 39 mm in diameter with enlarged lymph nodes in the ileal mesentery and a tumor of 24 mm in diameter in the rectum. Preoperative imaging studies suggested that the tumor in the ileal mesentery could be a low-grade malignant lymphoma, a plasmacytoma, a Castleman disease, an IgG4-related disease, a desmoid tumor, a carcinoid tumor, or a gastrointestinal stromal tumor. We performed laparoscopic low anterior resection for rectal cancer and biopsied an enlarged lymph node in the ileal mesentery to diagnose the tumor. He was diagnosed with rectal cancer with a lymph node metastasis (TNM classification 7th edition, pT3 pN1 cM0, and stage IIIB) and NET (G1) of the ileal mesentery. Although we considered complete resection for the NET (G1) lesion, it was a slow-growing tumor, and the 5-year survival rate of patients with gastrointestinal NET (91.3%) is better than that reported for rectal cancer stage IIIB (78.0%) [4, 5]. Thus, we felt that rectal cancer would determine the patient’s prognosis and decided to follow up the NET (G1) and prioritize adjuvant chemotherapy for the rectal cancer. He received oxaliplatin and capecitabine (XELOX) for 3 months as adjuvant chemotherapy. The NET (G1) lesion of the ileal mesentery had not expanded at follow-up. Three years later, anastomosis recurrence occurred, and we performed abdominoperineal resection of the rectal tumor. At the same time, we again considered resection of the NET lesion, but it had grown to 42 mm and involved both the supra mesenteric artery (SMA) and vein (SMV) and would be very difficult to remove. However, until this point, there were no occlusive bindings of the SMA and SMV. A CT scan that was taken 1 year after the surgery indicated multiple liver metastases and lymph node metastasis of the sacrum. Laboratory tests showed albumin 4.2 g/dl, creatinine 1.36 mg/dl, an international normalized ratio of 0.97, serum bilirubin 0.80 mg/dl, and an indocyanine green retention rate at 15 min of 5.9%. Regarding the tumor markers, serum carcinoembryonic antigen levels were elevated to 15.5 ng/ml. The serum carbohydrate antigen 19-9 and alpha-fetoprotein levels were within the respective normal ranges, and hepatitis B virus surface antigens and hepatitis C virus antibodies were negative. Contrast-enhanced abdominal computed tomography (CT) showed six masses in the right lobe of the liver and segment 3 and a tumor of 47 mm in diameter in the ileal mesentery. The masses in the liver showed ring-like enhancement (Fig. 1a), and the tumor in the mesentery showed heterogeneous enhancement in the arterial phase (Fig. 1b). [18F]-fluorodeoxyglucose positron emission tomography (FDG-PET) showed increased uptake by the masses in the liver and slight uptake by the tumor in the ileal mesentery (Fig. 1c, d). Magnetic resonance imaging (MRI) indicated four other masses in the right lobe of the liver and segment 4 which were not visible on CT and that had no accumulation of [18F]-FDG (Fig. 1e, f). All tumors expressed low intensity on T1-weighted MRI, high intensity on T2-weighted MRI, and high intensity on diffusion-weighted images (Fig. 2a–c). Gadolinium-ethoxybenzyl-diethylenetriamine pentaacetic acid-enhanced magnetic resonance imaging (EOB-MRI) revealed tumors with low signal intensity in the hepatocellular phase (Fig. 2d).

We suspected that the liver tumors were metastases from rectal cancer based on the imaging and laboratory test findings. To achieve hepatic clearance, right lobectomy with partial hepatectomy in segments 3 and 4 was necessary, and the functional future liver remnant (FLR) volume was 334 ml (26%). The postoperative residual hepatic tissue was insufficient to maintain normal physiological function. Therefore, we decided to achieve sufficient hypertrophy through the modified associating liver partition and portal vein ligation for staged hepatectomy (modified ALPPS) procedure, which has been introduced recently as a new surgical technique to increase FLR in patients with a marginal liver volume contemplating major liver resection [6]. The first stage of the surgery performed laparoscopically included cholecystectomy, ligation of the right portal vein, splitting of the hepatic tissue between the right and left lobe along the demarcation line, and partial hepatectomies of segments 3 and 4. No complications occurred, and the patient recovered uneventfully and was discharged on postoperative day 8. The liver volume increased rapidly after the first stage of the modified ALPPS. The FLR was 38.2% on day 11 and 41% on day 19 after the first stage. MRI performed on day 19 after the first stage showed a new tumor in segment 3. Therefore, right hepatic lobectomy and partial hepatectomy of segment 3 were performed on day 21 following the first stage of the modified ALPPS. The postoperative course was uneventful, and the patient was discharged on postoperative day 13.

**Fig. 3:**
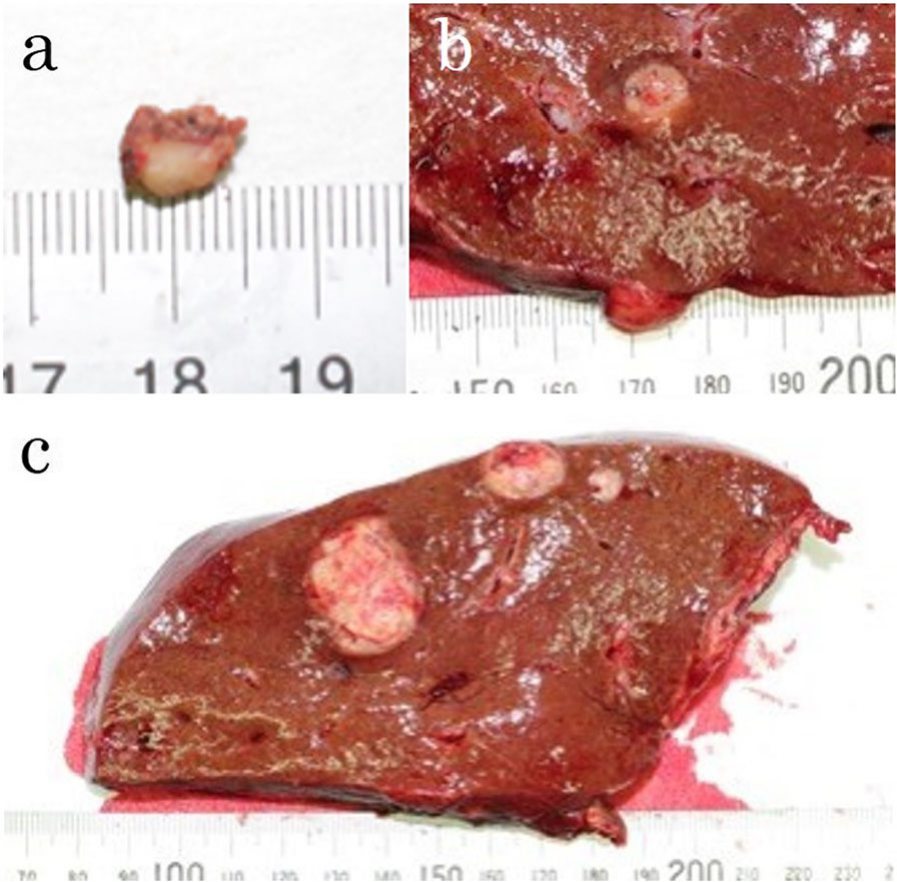
Multiple tumors of the resected specimen. **a** The tumor in segment 4 of the liver was a 5-mm well-defined yellowish-white elastic lesion. **b** The tumor in segment 6 of the liver was a 7-mm well-defined yellowish-white elastic lesion. **c** The tumor in segment 5 of the liver was a 25-mm well-defined yellowish-white elastic lesion

**Fig. 4:**
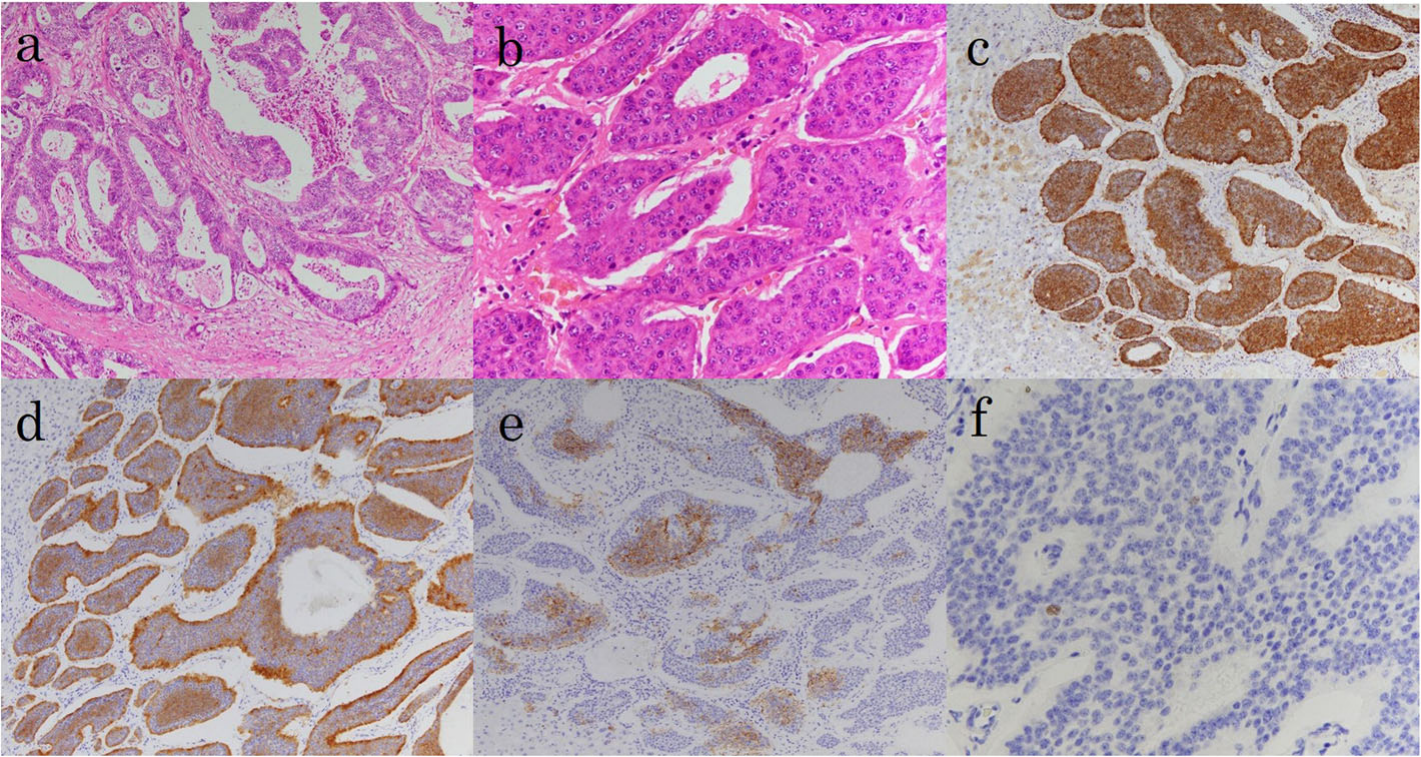
Histopathological examination of the multiple tumors. **a** All tumors except for two tumors in segments 4 and 6 were composed of well to moderately differentiated adenocarcinoma cells, growing in tubular or in a cribriform pattern. **b** The tumor in segment 6 showed a proliferation of atypical cells with rounded nuclei and eosinophilic cytoplasm that were arranged in a small nested pattern, accompanied by fibro-collagenous stroma. **c** Immunohistochemical examination of the tumor cells in segments 4 and 6 revealed that they were differentiation (CD) 56-positive. **d** Immunohistochemical examination of the tumor cells in segments 4 and 6 revealed that they were chromogranin-A-positive. **e** Immunohistochemical examination of the tumor cells in segments 4 and 6 revealed that they were synaptophysin-positive. **f** Immunohistochemical examination of the tumor cells in segments 4 and 6 revealed that the MIB-1 labeling index was less than 1%

Macroscopic examination of the cut specimen showed 16 masses, and all were well-defined yellowish-white elastic masses (Fig. 3a–c). Histopathological examination showed that 14 tumors located in the right lobe and segment 3 were composed of well to moderately differentiated adenocarcinoma cells growing in a tubular or cribriform pattern. The features resembled the previous specimens of rectal cancer, indicating metastatic recurrence of rectal adenocarcinoma (Fig. 4a). Two tumors located in segments 4 and 6 showed atypical cells with rounded nuclei and eosinophilic cytoplasm (Fig. 4b). Immunohistochemically, these atypical cells were positive for differentiation (CD) 56 (Fig. 4c), chromogranin-A (Fig. 4d), and synaptophysin (Fig. 4e). The MIB-1 labeling index was less than 1% (Fig. 4f). These features resembled those of the previous specimen, indicating metastatic recurrence of NET (G1). The patient underwent radiotherapy for lymph node metastasis of the sacrum and received oxaliplatin and capecitabine (XELOX) and was free from recurrence 7 months after the hepatectomy.

## Discussion

NET is a relatively rare tumor, but its incidence has increased over the past two decades due to improved awareness and diagnostic techniques and is currently estimated to occur in approximately 5.25 individuals per 100,000 [7]. NET is derived predominantly from enterochromaffin or Kulchitsky cells and has diverse pathological characteristics that typically correspond to the site of origin and hormone-secreting ability [8] and may develop in almost any organ. Most NETs occur in the gastrointestinal tract, pancreas, and bronchopulmonary system [2]. Primary mesenteric NETs are extremely rare, and there are only 11 reports of them, including our case [9–18]. Of these, 6 of the patients were male and 5 were female, and the patients ranged from 48 to 74 years of age (average, 64 years). Three of the patients had liver metastases, and 3 had SPMs of the sigmoid colon and rectal and prostate cancer.

The development of SPMs in patients with NET is a well-described phenomenon in Western nations. In the previous study involving a total of 9727 NET patients, 25.8% of NETs were associated with SPMs [3]. However, SPMs have been reported to occur in only 6% of patients with rectal NETs in Japan [19], and the frequency of SPMs with NETs differs between Japan and Western nations. A total of 80% of SPMs are recognized in the gastrointestinal tract, and the most common type of SPM is adenocarcinoma (49.4%) [20]. The pathogenesis of NETs associated with SPMs remains unclear but may be rooted in the tumorigenic properties of the various neuroendocrine peptides expressed and secreted by neuroendocrine cells. Peptides such as secretin, gastrin, bombesin, cholecystokinin, and vasoactive intestinal peptide are believed to promote the proliferation of tumor cells [3, 20]. Prognosis is affected by the progression of SPMs and the exacerbation of NET metastatic lesions rather than the primary lesion of NETs [21]. In our case, the patient had rectal cancer (pT3 pN1 cM0 and stage IIIB) and NET (G1) in the mesentery from the beginning. At first, we thought his prognosis was affected by rectal cancer and did not perform resection of the primary NET of the ileal mesentery.

One of the major prognostic factors of NETs that dramatically affect patient survival is the presence of liver metastases. It has been demonstrated that patients with liver metastases have a worse survival rate when compared with those without liver involvement [22]. Preoperative diagnosis of liver metastases from NETs with SPMs is extremely difficult using CT and MRI, as in our case. NET liver metastases show enhancement in the arterial phase of CT because most liver metastases are hypervascular. A total of 15% of these metastases may be seen only in the immediate arterial phase, and triple-phase multi-detector raw computed tomography (MDCT) or EOB-MRI is important in the initial evaluation of these lesions [23]. Seventy-five percent of NET cases show low intensity on T1-weighted MRI and high intensity on T2-weighted MRI [24]. These also correspond to the findings of liver metastasis of colorectal cancer. In our case, the liver metastasis of the NET in segment 6 did not show in the arterial phase of CT (Fig. 1e) but showed low intensity on T1-weighted MRI (Fig. 2a) and high intensity on T2-weighted MRI (Fig. 2b). Retrospectively, there was no distinction between the liver metastases of rectal cancer and NET on the images.

Liver metastasis of both colorectal cancer and NET (G1) is treated initially with surgical resection, if possible, and surgical resection of liver metastases from these sites has been demonstrated in terms of overall survival and quality of life [25, 26].

## Conclusions

We report a case of multiple liver metastases from synchronous double cancer of NET (G1) in the ileal mesentery and rectal cancer. Liver metastases from both the NET (G1) and rectal cancer were not distinguished in preoperative images of our case. Our findings suggest that it is necessary to consider the possibility of liver metastasis from NET (G1) when patients with synchronous double cancer of NET (G1) and gastrointestinal cancer have liver metastasis. Curative resection, precise pathological diagnosis, and adequately adjusted treatment may ensure a better prognosis.

## Data Availability

Not applicable.

## Abbreviations

ALPPS: Associating liver partition and portal vein ligation for staged hepatectomy
CT: Computed tomography
EOB-MRI: Gadolinium-ethoxybenzyl-diethylenetriamine pentaacetic acid-enhanced magnetic resonance imaging
FDG-PET: [18F]-fluorodeoxyglucose positron emission tomography
FLR: Functional future liver remnant
GI-NETs: Gastrointestinal neuroendocrine tumors
MDCT: Multi-detector raw computed tomography
MRI: Magnetic resonance imaging
NET: Neuroendocrine tumor
SPMs: Second primary malignancies
